# Early Psychosis and Trauma-Related Disorders: Clinical Practice Guidelines and Future Directions

**DOI:** 10.3389/fpsyt.2017.00033

**Published:** 2017-03-06

**Authors:** Casey A. Cragin, Martha B. Straus, Dawn Blacker, Laura M. Tully, Tara A. Niendam

**Affiliations:** ^1^Department of Clinical Psychology, Antioch University New England, Keene, NH, USA; ^2^CAARE Diagnostic and Treatment Center, Department of Pediatrics, University of California, Davis, Sacramento, USA; ^3^UC Davis Imaging Research Center, Department of Psychiatry and Behavioral Sciences, University of California, Davis, Sacramento, USA

**Keywords:** early psychosis, trauma, posttraumatic stress disorder, clinical practice guidelines, expert consensus method

## Abstract

Despite high rates of trauma-related disorders among individuals with early psychosis, no clinical practice guidelines for the treatment of comorbid early psychosis and trauma-related disorders exist to date. Indeed, the routine exclusion of individuals with past and current psychosis from participation in trauma research and practice has limited the accumulation of research that could support such clinical practice guidelines. While preliminary research evidence suggests that traditional, evidence-based treatments for trauma-related disorders can be safely and effectively employed to reduce symptoms of posttraumatic stress and chronic psychosis, it remains unclear whether such treatments are appropriate for individuals in the early stages of psychotic illness. Clinical experts (*N* = 118) representing 121 early psychosis programs across 28 states were surveyed using the expert consensus method. Forty-nine clinical experts responded and reached consensus on 46 of 49 expert consensus items related to the treatment of comorbid early psychosis and trauma-related disorders. Conjoint or family therapy and individual therapy were rated as treatment approaches of choice. Anxiety or stress management and psychoeducation were rated as treatment interventions of choice for addressing both trauma symptoms and psychotic symptoms. In addition, case management was rated as a treatment intervention of choice for addressing psychotic symptoms. No consensus was reached on expert consensus items regarding the appropriateness of a parallel treatment approach exposure interventions for addressing psychotic symptoms, or sensorimotor or movement interventions for addressing trauma symptoms. In areas where expert consensus exists and is supported by current research, preliminary clinical practice guidelines for the treatment of comorbid early psychosis and trauma-related disorders are offered. In areas where expert consensus does not exist, recommendations for future research are offered. The results of this study are intended to serve as a launching point for scientists and practitioners interested in advancing appropriate treatment for high-risk and underserved individuals with comorbid early psychosis and trauma-related disorders.

## Introduction

Rates of PTSD among individuals in the early stages of psychotic illness are high: in a study of Cincinnati psychiatric services, nearly 23% of first episode psychosis individuals presented with comorbid PTSD (1) versus an estimated 15% lifetime prevalence in individuals with chronic psychotic illness (2–4) and 6.8% prevalence in the general population (5). These high rates of comorbidity have prompted research on the effectiveness of interventions for comorbid psychosis and PTSD, with recent conceptual frameworks posing a reciprocal relationship between trauma and psychosis in the context of the cognitive model (6, 7). Empirically supported treatments for PTSD predominantly include trauma-focused treatments that provide direct exposure to traumatic events in order to combat the role of avoidance in the maintenance of PTSD (8, 9). Findings suggest these traditional evidence-based treatments for trauma-related disorders can be safely and effectively employed to reduce symptoms of posttraumatic stress and chronic psychosis (3, 10–12); however, it remains unclear whether such treatments are appropriate for individuals with early psychosis (i.e., within the first 5 years of illness onset). The early stages of psychotic illness are a critical period for intervention. With onset typically occurring between 15 and 25 years of age, psychosis symptoms disrupt important developmental trajectories in social, academic, and vocational domains. Combined with high rates of relapse (13) and comorbid trauma-related disorders (1), early intervention using evidence-based practices is necessary to prevent a long-term trajectory of accumulating disability (14). Yet, despite the burgeoning number of early psychosis treatment programs across the United States (15, 16), no clinical practice guidelines for treating comorbid early psychosis and trauma-related disorders currently exist.

The effect of exposure interventions on PTSD symptoms in adults with PTSD and chronic psychotic disorders has been explored in four studies to date. In an open trial study of 20 adults with PTSD and either schizophrenia or schizoaffective disorder, Frueh and colleagues (10) found cognitive behavioral therapy (CBT) that included imaginal exposure interventions integrated with treatment-as-usual (TAU) approaches significantly decreased PTSD symptoms and anger, as well as increased general mental health, compared to TAU alone. In another open pilot trial, van den Berg and van der Gaag (12) found eye movement desensitization and reprocessing (EMDR) plus TAU significantly decreased posttraumatic stress symptoms, psychotic symptoms (i.e., auditory verbal hallucinations and delusions), and other psychiatric symptoms (i.e., depression and anxiety), as well as increased self-esteem, after treatment compared to TAU alone. Similarly, in a within-group controlled study, de Bont and colleagues (3) found both prolonged exposure (PE) and EMDR decreased PTSD severity and diagnosis. Subsequently, in a randomized control trial, van den Berg and colleagues (17) found both PE and EMDR significantly decreased PTSD symptoms and diagnosis compared to waiting list (WL); however, PE, but not EMDR, resulted in full remission of PTSD compared to WL.

Two randomized control trials (RCTs) have examined the effect of CBT that did not include exposure interventions on PTSD symptoms in adults with PTSD and schizophrenia-spectrum disorders. Mueser and colleagues (11) found CBT integrated with TAU significantly decreased PTSD, mood, and anxiety symptoms, negative trauma-related cognitions, other psychiatric symptoms, and health-related concerns compared to TAU alone. Additionally, CBT participants endorsed increased knowledge of PTSD and client-case manager working alliance. The observed effects were significantly more robust for individuals with severe PTSD compared to those with mild or moderate PTSD. More recently, Steel and colleagues (18) found CBT without exposure, conducted in the context of TAU, did not significantly decrease trauma-related cognitions, severity of PTSD symptoms, positive symptoms of psychosis, severity of hallucinations and delusions, depression, or anxiety, or increase functioning or quality of life. Steel and colleagues concluded that exposure interventions focused on processing emotions related to traumatic memories may be needed in order for CBT to be effective in individuals with comorbid PTSD and psychotic disorders. Both studies also recommended future research include exposure interventions in treatment protocols in order to increase their effectiveness.

Collectively, these findings demonstrate that CBT, PE, and EMDR can be employed safely and effectively to reduce both PTSD and psychotic symptoms in adults with comorbid chronic psychosis and PTSD (3, 10–12); however, the inclusion of exposure interventions may result in more robust effects compared to non-exposure interventions in this population. There is, however, no available literature to guide the treatment of comorbid early psychosis and trauma-related disorders. As a result, additional research is needed to clarify which treatments are appropriate for individuals in the early stages of psychotic illness.

### The Current Study

Well-constructed clinical practice guidelines have the potential to improve the consistency, efficiency, value, and outcome of health care, as well as to empower patients and practitioners to make more informed health-care decisions, protecting both parties from the negative influences of uncertainty and antiquity; however, poorly constructed clinical practice guidelines have the potential to reduce the quality, efficiency, availability, and flexibility of health care (19). It is, therefore, important to use empirical methods to promote guideline development, ideally rooted in a strong foundation of carefully conducted RCTs on multiple large, independent, well-defined samples; however, RCTs often require years to conduct and the adoption of original research into clinical practice can take up to two decades (20); other methods can be used to generate preliminary guidelines and inform clinical practice in the interim.

One such method is the expert consensus method, which is designed to allow researchers to collect consensus evidence in cases where the outcome literature is unclear, incomplete, or absent, and must be supplemented with expert opinion (21). The expert consensus method has been used to develop clinical practice guidelines for dementia (22), obsessive–compulsive disorder (23), bipolar disorder (24), schizophrenia (25), PTSD (9), and complex PTSD (26). Here, we use the expert consensus method to develop preliminary clinical practice guidelines as a first step toward addressing the needs of individuals with comorbid early psychosis and trauma-related disorders.

We conducted a survey of clinical experts responsible for overseeing the clinical services provided in early psychosis programs across the United States. This survey elicited their expert opinions about treatment appropriateness for individuals with comorbid early psychosis and trauma-related disorders. In areas where expert consensus existed and was supported by current research, preliminary clinical practice guidelines for comorbid early psychosis and trauma-related disorders are offered. In areas where expert consensus does not exist, recommendations for future research are offered. The results of this study are intended to serve as a launching point for scientists and practitioners interested in advancing appropriate treatment for high-risk and underserved individuals with comorbid early psychosis and trauma-related disorders. To this end, the current study is designed to address the following research questions:

1. Which treatment modalities (e.g., individual therapy, conjoint or family therapy, consultation) are most appropriate for individuals with comorbid early psychosis and trauma-related disorders?
2. Which treatment approaches (e.g., single-diagnosis, sequenced, parallel, or integrated) are most appropriate?
3. Which treatment interventions are most appropriate for addressing psychotic symptoms? For treating trauma symptoms?
4. Is trauma-focused treatment appropriate? If so, under what clinical and psychosocial conditions (e.g., stage of psychosis, current psychosocial context, past psychosocial context)?
5. Should treatment modalities, approaches, or interventions be modified based on the individual’s developmental level (e.g., under age 18 or over age 25)? If so, how?
6. What are the barriers to treating comorbid early psychosis and trauma-related disorders in early psychosis programs?
7. How can the treatment of comorbid early psychosis and trauma-related disorders in early psychosis programs be improved?

## Materials and Methods

### Participants

Participants included clinical directors or persons in comparable positions responsible for overseeing the clinical services of early psychosis programs in the United States. Given the specialized nature of evidence-based care for early psychosis populations (14), these individuals were presumed to have the knowledge and experience needed to offer expert clinical opinions about the treatment of individuals with comorbid early psychosis and trauma-related disorders in the United States. There were no *a priori* exclusion criteria for this study.

### Procedures

Early psychosis program directories, located through an Internet search and consultation with experts in the field, were reviewed in order to identify potential recruitment sites (15, 16, 27, 28). This resulted in the identification of 121 early psychosis programs across 28 states (see Supplementary Material). Clinical directors or persons in comparable positions were identified for all 121 early psychosis programs in the United States. In some cases, one person fulfilled this role at multiple early psychosis programs. As a result, the total number of prospective participants (*N* = 118) was slightly lower than the total number of early psychosis programs (*N* = 121). No early psychosis programs or prospective participants were excluded.

Participants were recruited *via* email, including a brief description of the study and a link to the anonymous online survey. No identifying information was collected or attached to survey responses to allow the participants to respond as honestly as possible. Reminder emails were sent to all prospective participants 2 and 4 weeks after the initial recruitment email. Participants were offered two optional participation incentives: optional entry into a raffle for one of four $25 Amazon gift cards and optional receipt of study results. In order to ensure that survey responses remained anonymous, prospective participants were instructed to opt-in to one or both optional participation incentives by emailing the principle investigator with “RAFFLE” and/or “RESULTS” in the subject line. This study was reviewed and approved by the Institutional Review Board (IRB) of Antioch University New England (AUNE). The IRB of AUNE granted this study exempt status under 45 CFR 46.101(b)(2) exemption from 45 CFR part 46 requirements.

### Measure

Participants completed an anonymous 15–20 min online survey, administered *via* the Qualtrics secure web-based platform. The online survey contained 24–30 questions (the exact number varied depending on six conditional response questions) about participant characteristics, program characteristics, and expert consensus questions regarding modalities, approaches, interventions, treatments, developmental considerations, treatment barriers, and treatment improvements. Definitions of key terms and interventions were provided. See Supplementary Material for the complete survey and definitions.

The expert consensus questions were modeled after prior studies (22, 26, 29). Participants were instructed to use a 9-point scale to rate the appropriateness of modalities, approaches, interventions, and treatments. Scores in the 7–9 range indicate a degree of appropriateness, scores in the 4–6 range indicate a degree of equivocal opinion, and scores in the 1–3 range indicate a degree of inappropriateness with the following anchors: 9 = extremely appropriate: your modality, approach, intervention, or treatment of choice (TOC) (you may have more than one per question); 7–8 = appropriate: a first-line modality, approach, intervention, or treatment you would often use; 4–6 = equivocal: a second-line modality, approach, intervention, or treatment you would sometimes use (e.g., after first-line modalities, approaches, interventions, or treatments failed); 2–3 = usually Inappropriate: at most, a third-line modality, approach, intervention, or treatment you would rarely use; and 1 = extremely inappropriate: a modality, approach, intervention, or treatment you would never use.

### Data Analysis

Expert consensus data were analyzed using procedures identical to those described in Frances and colleagues (29). First, the mean and confidence interval (95%) was calculated for each expert consensus item. The confidence interval for each expert consensus item was used to assign a categorical rating based on the range into which the lowest confidence limit (LCL) fell. A categorical rating of first-line was assigned to modalities, approaches, interventions, and treatments with a LCL that fell into the 6.50–9.00 range; a categorical rating of second-line was assigned to modalities, approaches, interventions, and treatments with a LCL that fell into the 3.50–6.49 range; and a categorical rating of third-line was assigned to modalities, approaches, interventions, and treatments with a LCL that fell into the 1.00–3.49 range. The distribution of responses for each expert consensus item was then analyzed for consensus. The categorical ratings for each expert consensus item were coded (i.e., first-line = 1, second-line = 2, and third-line = 3), and a non-parametric chi-square test was conducted for each expert consensus item in order to determine whether or not expert consensus existed. Consensus was defined as when the response distribution of categorical ratings was statistically different from chance (*p* ≤ 0.05) (29). Finally, expert consensus items rated a 9 by 50% or more of participants were determined to represent a TOC for modalities, approaches, interventions, and treatments. Participant and program characteristics data are reported in Supplementary Material. Qualitative data were analyzed using a general inductive approach (30).

## Results

Online survey responses were collected from June 27 to August 5, 2016. Of the 118 clinical experts invited to participate, 66 (56%) responded. Seventeen (26%) of the 66 responses were omitted due to discontinuation of the survey prior to reaching the expert consensus items. The remaining 49 (42%) survey responses were included and analyzed to yield the following results.

### Quantitative Results

#### Participant Characteristics

Twenty-six (53%) participants identified a master’s degree and 23 (47%) identified a doctorate or professional degree as their highest level of completed education. Forty-three (88%) participants reported providing treatment to individuals with early psychosis and 35 (71%) reported providing treatment to individuals with comorbid early psychosis and trauma-related disorders in the last 12 months. Forty-nine (100%) participants reported receiving formal training or supervised clinical experience in the treatment of early psychosis: 35 (73%) reported receiving both formal training and supervised clinical experience, while 13 (27%) reported receiving formal training only. Thirty-nine (80%) participants reported also receiving formal training or supervised clinical experience in the treatment of trauma-related disorders: 23 (62%) reported receiving both formal training and supervised clinical experience, while 11 (30%) reported receiving formal training only and 3 (8%) reported receiving supervised clinical experience only. See Supplementary Material for specific early psychosis and trauma treatments in which participants reported receiving formal training and supervised clinical experience.

#### Program Characteristics

Participants represented early psychosis programs from 18 states (see Supplementary Material). Twenty-eight (57%) programs were based in the community, 11 (22%) programs were based in a university, and 2 (4%) programs were based in a hospital. The remaining 8 (16%) programs were based in a combination of community, hospital, or university settings. Thirty-eight (95%) programs served clients under age 18 and 28 (70%) served clients over age 25. Forty-two (86%) participants reported that their programs offered coordinated specialty care for early psychosis, the primary evidence-based model for outpatient treatment of early psychosis (14). See Supplementary Material for types of services offered.

Forty-eight (98%) programs provided staff members with formal training or supervised clinical experience in the treatment of early psychosis: 39 (85%) provided staff members with both formal training and supervised clinical experience, while 5 (11%) provided formal training only and 2 (4%) provided supervised clinical experience only. Twenty-three (47%) programs also provided staff members with formal training or supervised clinical experience in the treatment of trauma-related disorders: 33 (68%) provided staff members with both formal training and supervised clinical experience, while 7 (14%) provided formal training only and 9 (18%) provided supervised clinical experience only. See Supplementary Material for early psychosis and trauma treatments in which programs provided formal training and supervised clinical experience.

#### Treatment Modalities

Participants were asked to rate the appropriateness of individual therapy (i.e., seeing client alone), consultation (i.e., seeing family members or support persons alone), and conjoint or family therapy (i.e., seeing client and family members or support persons together) for clients aged 18–25 with comorbid early psychosis and trauma-related disorders. Conjoint or family therapy (LCL = 7.98), individual therapy (LCL = 7.73), and consultation (LCL = 6.87) were all rated as first-line treatment modalities. Conjoint or family therapy (TOC = 57.14%) and individual therapy (TOC = 53.06%), however, were rated as the treatments of choice. See Table 1 for expert consensus ratings of treatment modalities.

**Table 1:** **Expert consensus ratings of treatment modalities and approaches**.

				Expert ratings								
				Treatment of choice (TOC)		1st line		2nd line		3rd line		Total
	
	Lowest confidence limit (LCL)	M	SD	%	*N*	%	*N*	%	*N*	%	*N*	*N*
**Modality**												
Conjoint/family therapy	7.98^b^	8.31	1.14	57.14^a^	28	95.91	47	2.04	1	2.04	1	49
Individual therapy	7.73^b^	8.12	1.36	53.06^a^	26	91.83	45	6.12	3	2.04	1	49
Consultation	6.87^b^	7.39	1.81	30.61	15	79.59	39	14.28	7	6.12	3	49
**Approach**												
Integrated	7.40^b^	7.88	1.63	45.83	22	85.42	41	10.41	5	4.16	2	48
Sequenced^EP^	5.52^c^	6.04	1.79	4.17	2	41.67	20	54.16	26	4.16	2	48
Sequenced^TRD^	4.11^c^	4.73	2.14	2.08	1	20.83	10	50.00	24	29.16	14	48
Single-diagnosis^EP^	2.92^d^	3.52	2.08	2.08	1	10.41	5	33.33	16	56.25	27	48
Single-diagnosis^TRD^	2.60^d^	3.19	2.04	0.00	0	8.33	4	31.24	15	60.42	29	48
Parallel	4.29^nc^	5.00	2.46	6.25	3	31.25	15	41.67	20	27.09	13	48

*^a^TOC*.

*^b^First-line*.

*^c^Second-line*.

*^d^Third-line*.

*^nc^No consensus*.

*^EP^Early psychosis*.

*^TRD^Trauma-related disorder*.

#### Treatment Approaches

Participants were asked to rate the appropriateness of single-diagnosis (i.e., treating either early psychosis or trauma-related disorder only), sequenced (i.e., treating early psychosis before treating trauma-related disorder or *vice versa*), parallel (i.e., different providers treating early psychosis and trauma-related disorder at the same time), and integrated (i.e., the same provider treating early psychosis and trauma-related disorder at the same time) treatment approaches for clients aged 18–25 with comorbid early psychosis and trauma-related disorders. Integrated treatment (LCL = 8.10) was rated as a first-line treatment approach. Sequenced treatments, beginning with either the treatment of early psychosis (LCL = 5.52) or the treatment of the trauma-related disorder (LCL = 4.11), were rated as second-line treatment approaches. Single-diagnosis treatments, only treating early psychosis (LCL = 2.92) or the trauma-related disorder (LCL = 2.60), were rated as third-line treatment approaches. No consensus was reached on the appropriateness of parallel treatment. See Table 1 for expert consensus ratings of treatment approaches.

#### Treatment Interventions

Participants were asked to rate the appropriateness of treatment interventions for addressing either psychotic symptoms or trauma symptoms for clients aged 18–25 with comorbid early psychosis and trauma-related disorders. These various treatment interventions are often components of broader treatment protocols for early psychosis and/or trauma-related disorders. See Supplementary Material for definitions of interventions.

##### Psychotic Symptoms

Anxiety or stress management (LCL = 8.10), psychoeducation (LCL = 7.97), cognitive restructuring (LCL = 7.48), case management (LCL = 7.43), interpersonal effectiveness (LCL = 7.16), meditation or mindfulness (LCL = 6.66), and emotion-focused (LCL = 6.52) interventions were rated as first-line treatment interventions for addressing psychotic symptoms. Anxiety or stress management (TOC = 73.91%), psychoeducation (TOC = 73.91%), and case management (TOC = 50.00%) interventions were rated as treatment interventions of choice. Sensorimotor or movement (LCL = 4.86) and bilateral stimulation (LCL = 4.00) interventions were rated as second-line treatment interventions. No consensus was reached on the appropriateness of exposure interventions for addressing psychotic symptoms. See Table 2 for expert consensus ratings of treatment interventions for addressing psychotic symptoms.

**Table 2:** **Expert consensus ratings of interventions to address psychotic symptoms and trauma symptoms**.

				Expert ratings								
	
				TOC		1st line		2nd line		3rd line		Total
	LCL	M	SD	%	*N*	%	*N*	%	*N*	%	*N*	*N*
**Psychotic symptoms**												
Anxiety/stress management	8.10^b^	8.48	1.26	73.91^a^	34	95.65	44	2.17	1	2.17	1	46
Psychoeducation	7.97^b^	8.39	1.41	73.91^a^	34	89.13	41	8.70	4	2.17	1	46
Cognitive restructuring	7.48^b^	7.89	1.37	42.22	19	84.44	38	13.33	6	2.22	1	45
Case management	7.43^b^	7.89	1.54	50.00^a^	23	82.61	38	13.04	6	4.35	2	46
Interpersonal effectiveness	7.16^b^	7.60	1.45	32.56	14	79.07	34	18.61	8	2.33	1	43
Meditation/mindfulness	6.66^b^	7.18	1.74	31.11	14	68.89	31	26.66	12	4.44	2	45
Emotion-focused	6.52^b^	7.00	1.58	15.91	7	61.36	27	36.36	16	2.27	1	44
Sensorimotor/movement	4.86^c^	5.50	2.10	9.09	4	29.54	13	54.55	24	15.92	7	44
Bilateral stimulation	4.00^c^	4.60	1.91	4.76	2	9.52	4	69.04	29	21.42	9	42
Exposure	4.54^nc^	5.30	2.49	11.63	5	32.56	14	44.18	19	23.26	10	43
**Trauma symptoms**												
Anxiety/stress management	8.13^b^	8.49	1.18	68.89^a^	31	95.55	43	2.22	1	2.22	1	45
Psychoeducation	7.78^b^	8.20	1.41	61.36^a^	27	88.63	39	9.09	4	2.27	1	44
Meditation/mindfulness	7.45^b^	7.91	1.51	45.45	20	84.09	37	13.64	6	2.27	1	44
Cognitive restructuring	7.42^b^	7.83	1.32	42.86	18	85.72	36	11.90	5	2.38	1	42
Interpersonal effectiveness	6.99^b^	7.48	1.57	30.95	13	78.57	33	19.04	8	2.38	1	42
Emotion-focused	6.98^b^	7.40	1.35	20.93	9	74.42	32	25.58	11	0.00	0	43
Case management	6.81^b^	7.30	1.60	30.23	13	72.09	31	23.26	10	4.65	2	43
Exposure	6.00^c^	6.57	1.82	19.05	8	52.38	22	42.86	18	4.76	2	42
Bilateral stimulation	4.69^c^	5.44	2.30	12.82	5	30.77	12	53.85	21	15.38	6	39
Sensorimotor/movement	5.27^nc^	5.98	2.27	14.29	6	45.25	19	38.09	16	16.66	7	42

*^a^TOC*.

*^b^First-line*.

*^c^Second-line*.

*^nc^No consensus*.

##### Trauma Symptoms

Anxiety or stress management (LCL = 8.13), psychoeducation (LCL = 7.78), meditation or mindfulness (LCL = 7.45), cognitive restructuring (LCL = 7.42), interpersonal effectiveness (LCL = 6.99), emotion-focused (LCL = 6.98), and case management (LCL = 6.81) interventions were rated as first-line treatment interventions for addressing trauma symptoms. Anxiety or stress management (TOC = 68.89%) and psychoeducation (TOC = 61.36%) interventions were rated as treatments of choice. Exposure (LCL = 6.00) and bilateral stimulation (LCL = 4.69) interventions were rated as second-line treatment interventions. No consensus was reached on the appropriateness of sensorimotor or movement interventions for addressing trauma symptoms. See Table 2 for expert consensus ratings of treatment interventions for trauma symptoms.

#### Trauma-Focused Treatment

Trauma-focused treatment addresses exposure to traumatic events directly by asking clients to recall or encounter thoughts, images, feelings, or situations related to traumatic events. Participants were asked to rate the appropriateness of trauma-focused treatment for clients aged 18–25 with comorbid early psychosis and trauma-related disorders overall, at each stage of psychosis, and under specific current and past clinical and psychosocial conditions. Given that participants were previously asked to rate various treatment interventions that are often components of specific trauma-focused treatments, here, participants were asked to rate the appropriateness of trauma-focused treatment in general. Overall, trauma-focused treatment (LCL = 6.97) was rated as a first-line treatment for clients aged 18–25 with early psychosis and comorbid trauma-related disorders. See Table 3 for expert consensus ratings of trauma-focused treatment.

**Table 3:** **Expert consensus ratings of trauma-focused treatment**.

				Expert ratings								
				TOC		1st line		2nd line		3rd line		Total
	LCL	M	SD	%	*N*	%	*N*	%	*N*	%	*N*	*N*
**Overall**												
Trauma-focused treatment	6.97^a^	7.54	1.80	41.46	17	78.04	32	19.52	8	2.44	1	41
**Stage of psychosis**												
First-episode psychosis	7.21^a^	7.70	1.54	45.00	18	77.5	31	20.00	8	2.50	1	40
Genetic risk and deterioration	7.19^a^	7.70	1.60	35.00	14	87.5	35	10.00	4	2.50	1	40
Chronic/established psychosis	7.14^a^	7.65	1.59	35.00	14	87.5	35	10.00	4	2.50	1	40
Ultra-high risk/clinical high risk	7.07^a^	7.58	1.57	32.50	13	80	32	17.5	7	2.50	1	40
**Current condition**												
Attenuated or residual psychotic symptoms	7.12^a^	7.59	1.42	35.14	13	78.38	29	21.62	8	0.00	0	37
Other comorbid psychiatric disorder	6.35^b^	6.95	1.79	24.32	9	64.86	24	29.73	11	5.40	2	37
Comorbid personality disorder	6.29^b^	6.95	1.96	24.32	9	64.86	24	29.73	11	5.40	2	37
Low involvement of support persons	6.09^b^	6.73	1.92	29.73	11	54.06	20	40.55	15	5.41	2	37
Significant life stressors	6.00^b^	6.73	2.19	32.43	12	59.46	22	27.03	10	13.51	5	37
**Past condition**												
Multiple traumas	6.99^a^	7.63	1.94	42.11	16	86.84	33	7.89	3	5.26	2	38
Single trauma	6.71^a^	7.42	2.16	42.11	16	84.21	32	7.89	3	7.89	3	38
Long-duration symptoms	6.69^a^	7.25	1.65	22.22	8	74.99	27	22.22	8	2.78	1	36
Poor functioning	6.49^b^	7.05	1.68	21.62	8	67.57	25	29.73	11	2.7	1	37
Non-suicidal self-injury	6.38^b^	6.97	1.79	18.92	7	67.57	25	27.03	10	5.41	2	37
Severe symptoms	6.37^b^	7.03	1.95	22.22	8	69.45	25	25	9	5.56	2	36
Substance use	6.34^b^	6.89	1.66	18.92	7	62.16	23	35.14	13	2.7	1	37
Hospitalization	5.95^b^	6.72	2.28	27.78	10	63.89	23	25.01	9	11.12	4	36
High suicide risk	5.54^b^	6.32	2.36	24.32	9	48.65	18	37.83	14	13.52	5	37
High violence risk	5.14^b^	6	2.58	16.22	6	54.06	20	27.04	10	18.92	7	37

*^a^First-line*.

*^b^Second-line*.

##### Stage of Psychosis

Stages of psychosis included genetic risk and deterioration (i.e., family history of psychosis and decline in functioning without attenuated or threshold psychotic symptoms), ultra-high or clinical high risk (i.e., attenuated psychotic symptoms), first-episode psychosis (i.e., onset of threshold psychotic symptoms less than 5 years ago), and established or chronic psychosis (i.e., onset of threshold psychotic symptoms more than 5 years ago). Trauma-focused treatment (LCL = 6.97) was rated as a first-line treatment for clients at all stages of psychosis: first-episode psychosis (LCL = 7.21), genetic risk and deterioration (LCL = 7.19), chronic or established psychosis (LCL = 7.14), and ultra-high risk or clinical high risk (LCL = 7.07). See Table 3 for expert consensus ratings of trauma-focused treatment at each stage of psychosis.

##### Current Clinical and Psychosocial Conditions

Trauma-focused treatment was rated as a first-line treatment for clients with current attenuated or residual psychotic symptoms (LCL = 7.12). It was rated as a second-line treatment for clients with current comorbid personality disorders (LCL = 6.29), other comorbid psychiatric disorders (LCL = 6.35), low involvement of family members or support persons (LCL = 6.09), and significant life stressors (LCL = 6.00). See Table 3 for expert consensus ratings of trauma-focused treatment given current conditions.

##### Past Clinical and Psychosocial Conditions

Trauma-focused treatment was rated as a first-line treatment for clients with a history of multiple traumas (LCL = 6.99), single trauma (LCL = 6.71), and long-duration psychotic symptoms (LCL = 6.69). It was rated as a second-line treatment for clients with a history of poor functioning (LCL = 6.49) and severe psychotic symptoms (LCL = 6.37), as well as a history of hospitalization (LCL = 5.95), substance use (LCL = 6.34), non-suicidal self-injury (LCL = 6.38), high suicide risk (LCL = 5.54), and high violence risk (LCL = 5.14). See Table 3 for expert consensus ratings of trauma-focused treatment given past conditions.

### Qualitative Results

#### Developmental Considerations

Because the expert consensus items asked specifically about clients aged 18–25, participants who reported serving clients under age 18 or over age 25 were asked if and how the appropriateness of modalities, approaches, interventions, or treatments differ for these other age groups. Of those participants who reported serving clients under age 18 and over age 25, respectively, 19 (50%) and 6 (21%) agreed that the appropriateness of modalities, approaches, interventions, or treatments differs for the specified age group.

##### Family Involvement

Participants acknowledged both the ethical (e.g., consent) and supportive functions of the family, noting that they work harder to engage family members in treatment in general and in decision-making specifically when working with clients under age 18. One participant, for example, noted the increased importance of family consent and engagement for clients with comorbid early psychosis and trauma-related disorders due to the perception of “increased risk with trauma treatment.” In cases where family involvement is low, another participant reported wanting “to ensure the individual had […] other identified support persons.” For clients over age 25, participants noted alternative support persons like close friends or partners might be more apt to be involved in treatment than members of the client’s family of origin.

##### Modification of Treatment Materials or Interventions

Participants noted the importance of “using age appropriate materials, language, and consideration of developmental tasks.” Participants noted cognitive interventions might be less appropriate or require additional assessment or modification for clients under age 18 compared to older clients. In addition, participants noted the importance of skill building for clients under age 18. For example, one participant responded, “Ensure [the] young person has skills to manage [a] potential increase in symptoms prior to commencing trauma work.”

#### Treatment Barriers

Participants were also asked if they were aware of any barriers their early psychosis programs encountered in attempting to treat clients with comorbid early psychosis and trauma-related disorders and, if so, to describe those barriers. Twenty-eight (78%) participants reported being aware of such barriers.

##### Differentiating Trauma Exposure from Psychotic Experiences

Participants noted high endorsement of traumatic events and other stressful life experiences or difficulty determining whether traumatic events and other stressful life experiences were real or delusional. In addition, participants reported difficulty determining how to handle reports of the first episode of psychosis as a traumatic event. Participants described attempts to overcome these barriers by focusing on educating clients about stressful experiences in general and helping clients develop and utilize strategies to cope with stressful experiences in lieu of educating clients specifically about trauma or helping clients to directly process the reported traumatic events. One participant noted that inclusion of collateral information can help to clarify the validity of the experiences that are being reported.

##### Symptom Interference and Exacerbation

One participant, for example, noted impairment associated with either early psychosis or trauma-related disorders can impede recovery from the other disorder. This participant described trauma as a major source of stress that can worsen psychotic symptoms. Another participant noted that psychotic symptoms interfere with the processing of traumatic events, especially in cases where the first episode of psychosis was experienced as traumatic.

##### Inadequate Training and Supervision

One participant noted that programmatic training, as well as available tools and interventions, focus only on early psychosis treatment despite a clearly identified need to be able to integrate early psychosis and trauma treatment. This participant also noted individual efforts to obtain training in trauma treatments on the part of clinicians have not been effective due to a lack of structured supervision and technical support. Another participant noted programmatic efforts to provide training in trauma treatments have not been effective due to a lack of available funding.

##### Discomfort Treating Both Trauma and Psychosis

Some participants reported not treating clients with comorbid trauma-related disorders due to specializing in early psychosis. In contrast, one participant noted clients are often misdiagnosed in the community as a result of practitioners specializing in trauma treatment incorrectly conceptualizing psychotic symptoms as trauma symptoms. Another participant noted difficulty identifying referral sites that are comfortable providing both early psychosis and trauma treatment.

#### Improving Treatment

Finally, participants were asked to provide any additional information they thought would help to improve the treatment of clients with comorbid early psychosis and trauma-related disorders. Eleven (22%) participants offered such suggestions.

##### Increase Training in Trauma Assessment and Treatment

Participants suggested increased training in trauma assessment and treatment would improve the treatment of clients with comorbid early psychosis and trauma-related disorders. For example, one participant noted, “We have addressed a lot of training but never trained in the context of comorbidity with trauma and psychosis.” Another participant suggested “early identification of trauma or stressful experiences using a[n] evidence-based scale to evaluate need for further treatment.”

##### Increase Trauma Research and Treatment Planning Guidance

Participants also suggested increased trauma research in general and related to treatment planning in particular. For example, one participant responded, “I wish that there were more data comparing treatments to guide decisions about what treatment options would be best for a specific individual.” Another participant noted a need for greater consistency in how trauma-related disorders are treated in early psychosis programs; however, participants noted that research-based treatment protocols would also need to allow treatment to be tailored to client symptoms and client and family preferences.

##### Increase Funding for Multidisciplinary Programs

Finally, participants suggested increased funding for programs that treat clients with a wider range of presenting problems including early psychosis, rather than for programs that specialize in treating early psychosis only, would improve the treatment of clients with comorbid early psychosis and trauma-related disorders.

## Discussion

With the growing number of early psychosis programs in the United States and abroad, this study represents an essential first step toward addressing the needs of individuals with comorbid early psychosis and trauma-related disorders. The development of clinical practice guidelines has been limited historically by the routine exclusion of individuals with past and present psychosis from participation in trauma research and practice (31–33), as well as trauma symptoms not being adequately addressed in psychosis research and practice. Using a comprehensive online survey of clinical experts who are responsible for overseeing the clinical services provided in early psychosis programs, we investigated the current opinions and intervention practices that are guiding the treatment of individuals with comorbid early psychosis and trauma-related disorders in the United States. Based on these responses and preliminary evidence that traditional evidence-based treatments for trauma-related disorders can be safely and effectively employed to reduce symptoms of posttraumatic stress and chronic psychosis (3, 10–12), we offer preliminary clinical practice guidelines and recommendations for future research.

### Preliminary Clinical Practice Guidelines and Suggestions for Future Research

#### Selecting a Treatment Modality

More than half of the clinical experts surveyed in this study rated conjoint or family therapy and individual therapy as their treatment modalities of choice when working with individuals with comorbid early psychosis and trauma-related disorders. This suggests that practitioners should see the client and family members or alternative support persons together with client consent or see the client alone at the start of treatment. This is consistent with current treatment guidelines for psychosis, which support the use of individualized and integrated family interventions (34–36). Additionally, the clinical experts surveyed in this study believed involving family members in the client’s treatment is particularly important for individuals under age 18; however, practitioners should also consider the benefits of involving alternative support persons, such as friends or romantic partners, when other family involvement is low or when treating individuals over age 25.

Consultation with family or support persons (without the client present) was also rated as a first-line treatment modality. In cases in which conjoint or family therapy or individual therapy is ineffective, seeing family members or alternative support persons alone with client consent could be an appropriate alternative treatment modality. Family members often experience significant burden when caring for individuals with serious mental illness (37). Therefore, family support may be helpful in protecting the family system if the client refuses to engage; however, current practice models suggest that engagement of family alone would likely not be sufficient in promoting recovery in the client (38).

#### Selecting a Treatment Approach

Integrated treatment was rated as a first-line treatment approach and sequenced treatments were rated as second-line treatment approaches for the treatment of comorbid early psychosis and trauma-related disorders. The first-line rating of an integrated treatment approach is somewhat surprising given that inadequate training and supervision, as well as inadequate institutional and financial support, were cited as barriers to integrated treatment in these open response data. Integrated treatment may represent the ideal approach that clinical experts recognize as most appropriate and often strive to provide even if they are ill equipped to do so. Funding should be provided to develop innovative programs that strive to address the complex needs of the early psychosis population through staff training and additional program supports.

The second-line rating of sequenced treatment approaches is consistent with participants’ report that the exacerbation of early psychosis by a comorbid trauma-related disorder, or visa versa, is a potential barrier to treatment. Clinical experts gave examples of psychotic symptoms interfering with or worsening as a result of treatment of a comorbid trauma-related disorder, as well as the traumatic nature of psychotic symptoms for some clients interfering with treatment of early psychosis. In such cases, sequenced treatment (i.e., treating the exacerbating disorder first in part or in entirety) may be more appropriate than integrated treatment. Practitioners who elect to use a sequenced treatment approach, however, should clearly delineate the client’s treatment goals and carefully monitor client progress in the initial phase of treatment. A sequenced approach carries with it the risk that treatment will ultimately focus disproportionately on a single-diagnosis if the provider never feels the client is stable enough to shift to the second phase of treatment (39). Importantly, single-diagnosis treatment was rated as a third-line treatment approach, indicating that the clinical experts surveyed in this study believe that treating only early psychosis or only a trauma-related disorder when both conditions are present is inappropriate.

No consensus was reached regarding the appropriateness of a parallel treatment approach. This is consistent with current recommendations that suggest coordinating parallel treatment by different providers, often in different treatment settings, may fail to address the overlapping aspects of the comorbid psychiatric conditions or work at cross-purposes (39). Parallel treatment may, however, have merits in addressing barriers related to practitioner discomfort treating both trauma and early psychosis. Successful parallel treatment may be possible in the context of the early psychosis coordinated specialty care model, in which multiple practitioners from different specialties work together as a team to address the various needs of early psychosis clients (14). If practitioners specializing in the treatment of trauma-related disorders were added to these multidisciplinary teams, clients could access appropriate treatment for trauma-related disorders without compromising their access to evidence-based early psychosis care. In addition, working within a multidisciplinary team with specialty in early psychosis care would likely increase the competence and comfort of these trauma specialists with treating clients with early psychosis.

#### Selecting Treatment Interventions to Address Psychotic and Trauma Symptoms

First-line treatment interventions for addressing both psychotic and trauma symptoms included: anxiety or stress management, psychoeducation, case management, cognitive restructuring, emotion-focused interventions, interpersonal effectiveness, and meditation or mindfulness interventions. Bilateral stimulation was rated as a second-line treatment intervention for addressing both psychotic and trauma symptoms. When addressing trauma symptoms in the context of psychosis, exposure interventions were rated as second-line interventions. Sensorimotor or movement interventions were also rated as second-line treatment interventions for addressing psychotic symptoms.

Based on these ratings and consistent with evidence-based cognitive behavioral models for treating psychosis (35, 40) and PTSD (8, 9), we make the following recommendations. Practitioners should begin by providing psychoeducation about early psychosis and trauma. This should include descriptions of psychotic and trauma symptoms and information about treatment rationale and efficacy in order to help the client and their support persons understand the client’s problems as surmountable over time with appropriate treatment. Practitioners should then use anxiety and stress management interventions to help individuals develop coping skills to reduce stress and stress-related difficulties. Throughout treatment, practitioners should also provide case management to coordinate services and identify resources needed by the client. Finally, practitioners should select from first-line interventions (e.g., cognitive restructuring, emotion-focused, interpersonal effectiveness, and meditation or mindfulness) to address any residual psychotic and trauma symptoms.

#### Using Trauma-Focused Treatments and Exposure

Clinical experts surveyed here rated trauma-focused treatment (i.e., treatments that address exposure to traumatic events directly by asking individuals to recall or encounter thoughts, images, feelings, or situations related to traumatic events) as a first-line treatment for individuals aged 18–25 with comorbid early psychosis and trauma-related disorders in general and at all stages of psychosis. Trauma-focused treatment was rated as a first-line treatment for individuals presenting with current attenuated or residual psychotic symptoms, as well as a history of both single and multiple traumas and long-duration psychotic symptoms.

Furthermore, trauma-focused treatment was rated as a second-line treatment for individuals presenting with additional comorbidities and complexities, including: comorbid personality disorders, low involvement of support persons, significant life stressors, as well as a history of poor functioning, severe psychotic symptoms, substance use, non-suicidal self-injury, high suicide risk, high violence risk, and/or hospitalization. This indicates that the clinical experts surveyed in this study believe trauma-focused treatment may be appropriate for such individuals if more appropriate alternatives, not explored in this study, have failed. Notably, there were no current or past conditions for which trauma-focused treatment was rated as inappropriate. Practitioners should, therefore, diligently monitor areas of risk when utilizing trauma-focused treatment with individuals with comorbid early psychosis and trauma-related disorders; however, these risk factors should not be viewed as contraindications for trauma-focused treatment.

These findings contradict the experts’ ratings of exposure and bilateral stimulation, two interventions considered to be key components of trauma-focused treatments, as second-line interventions. It may be the case that clinical experts believe trauma-focused treatments that include interventions of choice (e.g., psychoeducation, anxiety, and stress management) and first-line interventions (e.g., cognitive restructuring, emotional-focused) before or in addition to exposure, for example, are more appropriate for individuals with comorbid early psychosis and trauma-related disorders than exposure interventions alone. Conversely, clinical experts may worry about the possible negative impact of exposure interventions on the recovery process, such as the exacerbation of psychotic symptoms, and may hesitate to use them. To date, the positive effect of exposure interventions on chronic psychotic symptoms in adults has been reported as effective in one published study (12), and two other studies have noted that the exclusion of exposure may have decreased the observed effectiveness of their treatment protocols (11, 18).

Exposure interventions are a primary component of all cognitive and behavioral interventions, including cognitive behavioral therapy for psychosis (CBTp), the early psychosis treatment in which participants received and programs provided training and supervision most often. It is possible that clinical experts are not utilizing recommended exposure components of CBTp in their practice despite evidence that doing so is beneficial, which is a phenomenon commonly seen in trauma treatment as well (31). It is also possible that clinical experts are utilizing exposure interventions without recognizing they are doing so, including psychoeducation about psychotic or trauma symptoms and behavioral experiments. This study asked participants to rate the appropriateness of component interventions, as opposed to combinations of interventions, in order to guide the composition of treatment for comorbid early psychosis and trauma-related disorders. Had participants been asked to consider the use of exposure in the context of broader treatment approaches (e.g., CBT), they might have responded more favorably to exposure interventions.

While the clinical experts surveyed in this study were unable to agree on the appropriateness of exposure interventions for addressing psychotic symptoms in individuals with comorbid trauma-related disorders, they agreed that exposure interventions are appropriate for addressing trauma symptoms in this population when first-line interventions have proved to be ineffective. Nonetheless, because exposure is such an important part of trauma treatment, expert attitudes toward exposure therapy for this population merit greater exploration. Research is needed to determine whether practitioners are either not utilizing or not recognizing their use of exposure interventions. Similarly, future studies should determine which exposure interventions are considered most appropriate when treating individuals with early psychosis in general, versus individuals with comorbid early psychosis and trauma-related disorders in particular.

#### Using Sensorimotor and Movement Interventions to Address Trauma Symptoms

Sensorimotor and movement interventions were included in the current study to reflect the wide range of evidence-based interventions used in clinical practice, despite not being included in the expert consensus study of PTSD conducted over a decade ago (9) and being rated as second-line treatment interventions in the expert consensus study of complex PTSD. Sensorimotor and movement interventions are used to assist individuals with trauma-related disorders regulate their autonomic nervous system, think more clearly, and derive information from emotional states more accurately by processing dissociated, incomplete, or ineffective sensorimotor reactions (e.g., trauma-related images, sounds, smells, and physical sensations) (41). Individuals with early psychosis are frequently vulnerable to excessive autonomic arousal in response to stress (42) and may misinterpret anomalous cognitive or perceptual experiences resulting in emotional arousal and behavioral withdrawal (7) secondary to trauma exposure (43). While mind-body interventions, like sensorimotor and movement interventions, are important to contemporary trauma treatment, their utility for the treatment of psychosis alone has not been well investigated (44). The experts’ rating of these approaches as second-line for the treatment of psychotic symptoms may represent growing interest in the integration of mind-body practices into psychosis care, but more well-controlled studies are needed before conclusions can be drawn (45). Currently, these approaches are not seen as core interventions for individuals with psychosis (34). Therefore, additional research is needed to determine whether sensorimotor and movement interventions could be beneficial for individuals with only early psychosis, as well as for individuals with comorbid early psychosis and trauma-related disorders.

#### Understanding Reports of Trauma in the Context of Psychosis

Open response data indicated that practitioners are often concerned about the validity of high rates of trauma exposure and other stressful life experiences reported by individuals with comorbid early psychosis and comorbid trauma-related disorders, particularly when trauma-related content is mixed with delusional content. As a result, clinical experts surveyed in this study reported program-wide efforts to address this issue by focusing on psychoeducation about stress in general and on developing and using coping skills to manage stress in daily life rather than providing psychoeducation about trauma and processing traumatic events. Auditory hallucinations and non-bizarre delusions of guilt, paranoia, or persecution occur in up to 40% of individuals with severe PTSD (46, 47). The content of these psychotic symptoms are often, though not always, trauma-related (48), and the relationship between trauma and psychosis is extraordinarily complex both causally and diagnostically (42, 43). Future research should aim to provide clearer guidance on how to safely and effectively address the mixture of trauma-related content and the content of delusions/hallucinations in treatment. In the meantime, practitioners should obtain collateral information to understand the temporal relationship between reported traumatic events and psychotic symptom development and conceptualize psychotic symptoms with trauma-related content as an indication that trauma-focused treatment, including psychoeducation about trauma, may be appropriate.

### Limitations

A limitation of all studies utilizing the expert consensus method is that the opinion of experts may be wrong (29). As a result, it is recommended that practitioners consider the results of this study in conjunction with the results of existing and emerging literature on the treatment of comorbid early psychosis and trauma-related disorders. In addition, the outcomes associated with implementation of these preliminary clinical practice guidelines should be evaluated to determine whether they are efficacious and effective.

Additionally, the survey utilized in this study was anonymous to encourage participants to respond as honestly as possible about their personal and programmatic clinical decision-making and intervention practices in the course of treating individuals with comorbid early psychosis and trauma-related disorders. As a result, we were not able to evaluate potential differences between those individuals who were contacted and responded versus those who did not respond.

Finally, the response rate for this study (42%) is lower than the typical response rates of other expert consensus method studies; however, the number of participants included in this study (*N* = 49) is comparable (29). While the expert consensus method is appropriate for use with clinical experts, it has been used primarily with preselected groups of research experts, which tends to increase the response rate (29). To yield the largest possible sample of respondents for this study, we consulted multiple published resources and available clinical and research experts and invited individuals from across the United States to participate. As research in this area increases, future endeavors to develop more comprehensive practice guidelines should include clinical and research experts in psychosis, as well as trauma, treatment to incorporate a variety of perspectives and sources of knowledge.

## Conclusion

This study addresses a gap in the existing outcome literature on the treatment of comorbid early psychosis and trauma-related disorders by supplementing it with consensus evidence obtained from a national survey of clinical experts. The clinical experts reached consensus on 46 (94%) of the 49 expert consensus items. In areas where expert consensus existed, preliminary clinical practice guidelines for comorbid early psychosis and trauma-related disorders were offered. Recommendations for future research were also proposed in areas in which expert consensus did not exist.

Perhaps most important is what this study did not find: the clinical experts surveyed in this study did *not* rate the use of trauma-focused treatment, or any component intervention including exposure interventions, as inappropriate for individuals with comorbid early psychosis and trauma-related disorders under any condition. In contrast, the clinical experts agree that not treating early psychosis and trauma-related disorders when both conditions are present is inappropriate. As a result, practitioners should use existing research evidence, clinical expertise and judgment, and client preferences and values to treat comorbid early psychosis and trauma-related disorders in individuals presenting with both conditions (49).

## Author Contributions

CC contributed to conception and design of the study, acquired, analyzed, and interpreted these data, and drafted and revised the manuscript. MS, DB, and LT contributed to conception and design of the study, as well as to revisions of the manuscript. TN contributed to conception and design of the study, acquisition and interpretation of these data, and revisions of the manuscript. All authors approved the final version of the manuscript to be published. In addition, all authors agreed to be accountable for all aspects of the work, including ensuring that all questions related to the accuracy and integrity of the work are appropriately investigated and resolved.

## Conflict of Interest Statement

This research was conducted in the absence of any commercial or financial relationships that could be construed as a potential conflict of interest.
