# Supplementary material for: Early Psychosis and Trauma-Related Disorders: Clinical Practice Guidelines and Future Directions
Source: Front Psychiatry. 2017 Mar 6;8:33. doi: 10.3389/fpsyt.2017.00033 (PMC5337515; doi:10.3389/fpsyt.2017.00033)
Supplement: Supplementary file 1 [file data_sheet_1.docx]

# Supplementary Material

Early Psychosis and Trauma-Related Disorders:

Clinical Practice Guidelines and Future Directions

Cragin, C. A.*, Straus, M. B., Blacker, D., Tully, L. M., and Niendam, T.A.

# Expert Consensus Survey of Early Psychosis Programs

**Purpose of the Study**

As the clinical director (or other person who is responsible for overseeing the clinical services) of an early psychosis program in the United States, you are being asked by a researcher at Antioch University New England to complete an online survey. The researcher wants to know your expert opinion on *how to best treat clients with early psychosis (i.e., onset of threshold psychotic symptoms occurring less than 5 years ago) and comorbid trauma-related disorders (i.e., posttraumatic stress disorder or other psychiatric disorder resulting from exposure to one or more traumatic event)*. The researcher will use the results of the online survey to inform clinical practice guidelines and recommendations for future research related to the treatment of clients with early psychosis and trauma-related disorders. The online survey consists of 24-30 questions and takes about 15-20 minutes to complete.

## Confidentiality

No identifying information will be collected or attached to survey responses. Survey responses will be stored in a password-protected, electronic database. Only researchers directly involved in this study will have access to survey responses. Study results will be reported only as data that summarizes the responses of all participants.

## Benefits of Participation

Your participation may benefit the field of psychology or clients with early psychosis and trauma-related disorders indirectly by increasing the knowledge base on how to best treat clients with early psychosis and trauma-related disorders.

Your participation may benefit you directly in up to two ways: as a prospective participant, you are eligible for up to two optional participation incentives including (1) entry into a raffle for one of four $25 Amazon gift cards and (2) receipt of a copy of the results of this study. To opt-in to one or both of these optional participation incentives, please email the researcher at [ccragin@antioch.edu](mailto:ccragin@antioch.edu) with “RAFFLE” and/or “RESULTS” in the subject line.

## Risks of Participation

There are no known risks to participating in this study.

## Voluntary Nature of the Study

You may skip any questions you are not comfortable answering or exit the survey at any time; however, please answer as many questions as you can. If you choose not to complete the survey, there will be no penalty and it will not affect your eligibility for optional participation incentives.

## Contacts

If you have any questions about the study or survey, you may contact the Principal Investigator, Casey A. Cragin, M.S., by telephone at (617) 653-9762 or by email at [ccragin@antioch.edu](mailto:ccragin@antioch.edu) or the Chair of the Dissertation Committee, Martha B. Straus, Ph.D., by telephone at (603) 283-2187 or by email at [mstraus@antioch.edu](mailto:mstraus@antioch.edu).

If you have any questions about your rights as a research participant, you may contact the Chair of the Institutional Review Board Committee, Kevin P. Lyness, Ph.D., by telephone at (603) 283-2149 or by email at [klyness@antioch.edu](mailto:klyness@antioch.edu) or the Vice President of Academic Affairs, Melinda Treadwell, Ph.D., by telephone at (603) 283-2444 or by email at [mtreadwell@antioch.edu](mailto:mtreadwell@antioch.edu).

Please print and retain a copy of this informed consent document for your records.

I have read and understood the information provided above. I **AGREE** to participate in this study and wish to be directed to the beginning of the survey.

I **DO NOT AGREE** to participate in this study and wish to exit the survey.

**For the purposes of this survey, the questions below refer to the following key terms:**

**Early Psychosis** — Onset of threshold psychotic symptoms less than 5 years ago

**Psychotic Symptoms** — Delusions (e.g., paranoid, grandiose, or somatic ideas or beliefs that are firmly held despite contrary evidence), hallucinations (e.g., auditory, visual, somatic, olfactory, or gustatory perceptions in the absence of corresponding external stimuli), or disorganized communication (e.g., losing track of or jumping around from one topic to another in conversation; behaving in ways that do not fit the situation)

**Trauma-Related Disorder** — Posttraumatic stress disorder (PTSD) or other psychiatric disorder resulting from exposure to one or more traumatic event

**Traumatic Event** — A situation (e.g., child physical, sexual, emotional, or psychological abuse; child neglect; domestic, school, or community violence; natural disasters like fire, tornado, floor, or hurricane; vehicular or other serious accident; war, terrorism, or refugeeism; medical trauma; and traumatic grief like sudden and/or violence death of a loved one) in which an individual is exposed directly or indirectly (e.g., witnessing, learning about) to actual or threatened death, serious injury, or sexual violence

**Trauma Symptoms** — Re-experiencing or intrusion symptoms (e.g., recurrent, involuntary, or distressing thoughts or images of the traumatic event), avoidance symptoms (e.g., avoidance of thoughts of, feelings about, and reminders associated with the traumatic event), negative cognitions or mood (e.g., persistent, distorted, or exaggerated negative beliefs about self, others, world, or cause or consequences of the traumatic event accompanied by negative emotions or inability to experience positive emotions), and hyperarousal symptoms (e.g., impaired emotional, behavioral, or cognitive regulation)

Please click here to download these definitions of key terms for your reference.

**Please keep these definitions in mind as you answer the questions below.**

Q1 What is the highest level of education you have completed? Please specify your discipline (e.g., psychiatry, psychology, social work, marriage and family therapy) in the space provided.

- Doctorate (e.g., PhD, PsyD, EdD) or Professional Degree (e.g., MD) (please specify discipline) [Small Text Box]
- Master's Degree (e.g., MA, MSW, MFT) (please specify discipline) [Small Text Box]
- Bachelor's Degree (e.g., BA, BS) (please specify discipline) [Small Text Box]
- Associate’s Degree (e.g., AA, AS) (please specify discipline) [Small Text Box]
- Other (please specify) [Small Text Box]

Q2 Have you personally treated a client with **early psychosis** in the last 12 months?

- Yes
- No

Q3 Have you personally treated a client with **early psychosis and a comorbid trauma-related disorder** in the last 12 months?

- Yes
- No
- Unknown

Q4 Have you personally received formal training and/or supervised clinical experience in the treatment of **early psychosis**?

- Yes, formal training and/or supervised clinical experience
- No
- Unknown

If Yes Is Not Selected, Then Skip To Q6

Q5 Please **LIST** the treatments for **early psychosis** in which you have received formal training and/or supervised clinical experience:

- Formal Training: [Medium Text Box]
- Supervised Clinical Experience: [Medium Text Box]

Q6 Have you personally received formal training and/or supervised clinical experience in the treatment of **trauma-related disorders**?

- Yes, formal training and/or supervised clinical experience
- No
- Unknown

If Yes Is Not Selected, Then Skip To Q8

Q7 Please **LIST** the treatments for **trauma-related disorders** in which you have received formal training and/or supervised clinical experience:

- Formal Training: [Medium Text Box]
- Supervised Clinical Experience: [Medium Text Box]

Q8 In what state is your early psychosis program located?

- Arizona
- California
- Colorado
- Connecticut
- Florida
- Georgia
- Illinois
- Indiana
- Iowa
- Maine
- Maryland
- Massachusetts
- Michigan
- Minnesota
- Mississippi
- Missouri
- Montana
- New Mexico
- New York
- North Carolina
- Ohio
- Oklahoma
- Oregon
- Pennsylvania
- Texas
- Utah
- Virginia
- Washington

Q9 In what type of setting is your early psychosis program based? Please select all that apply.

- Community
- Hospital
- University
- Other (please specify) [Small Text Box]

Q10 What age range does your early psychosis program serve?

Minimum age of clients served [Small Text Box]

Maximum age of clients served [Small Text Box]

Q11 **Coordinated Specialty Care (CSC)** *is a recovery-oriented treatment program for clients with early psychosis. A team of specialists work with the client, involving family members or support persons as much as possible, to create a personal treatment plan and offer psychotherapy, medication management, family education and support, and education and employment support services. Examples of CSC programs in the United States include NAVIGATE, the Connection Program, OnTrackNY, the Specialized Treatment Early in Psychosis (STEP) Program, and the Early Assessment and Support Alliance (EASA).*

Does your early psychosis program offer Coordinated Specialty Care for early psychosis?

- Yes
- No
- Unknown

Q12 Which of the following services does your early psychosis program offer? Please select all that apply.

- Comprehensive Assessment
- Case Management
- Medication Management
- Group Psychotherapy
- Individual Psychotherapy
- Family Psychoeducation and Support
- Supported Education and/or Employment Services
- Care Coordination (e.g., regular meetings of clinical team to coordinate services provided by all clinic staff)
- Other (please specify) [Medium Text Box]

Q13 Does your clinical staff receive formal training and/or supervised clinical experience in the treatment of **early psychosis**?

- Yes, formal training and/or supervised clinical experience
- No
- Unknown

If Yes Is Not Selected, Then Skip To Q15

Q14 Please **LIST** the treatments for **early psychosis** in which your clinical staff receives formal training and/or supervised clinical experience:

- Formal Training: [Medium Text Box]
- Supervised Clinical Experience: [Medium Text Box]

Q15 Does your clinical staff receive formal training and/or supervised clinical experience in the treatment of **trauma-related disorders**?

- Yes, formal training and/or supervised clinical experience
- No
- Unknown

If Yes Is Not Selected, Then Skip To Instructions [Before Q17]

Q16 Please **LIST** the treatments for **trauma-related disorders** in which your clinical staff receives formal training and/or supervised clinical experience:

- Formal Training: [Medium Text Box]
- Supervised Clinical Experience: [Medium Text Box]

For Questions 17-20, you will be asked to **rate the appropriateness of different treatment modalities, approaches, and interventions for clients age 18-25 with early psychosis and a comorbid trauma-related disorder**. If your early psychosis program serves clients under age 18 or over age 25, you will have an opportunity to share how your responses would differ for these age groups later in the survey.

**For each item, use scores in the 7-9 range to indicate a degree of appropriateness, scores in the 4-6 range to indicate a degree of equivocal opinion, or scores in the 1-3 range to indicate a degree of inappropriateness**, with the following anchor points:

**9** = **Extremely Appropriate**: Your modality, approach, or intervention of choice (you may have more than one per question)

**7-8** = **Appropriate**: A first-line modality, approach, or intervention you would often use

**4-6** = **Equivocal**: A second-line modality, approach, or intervention you would sometimes use (e.g., after first-line modalities, approaches, or interventions failed)

**2-3** = **Usually Inappropriate**: At most, a third-line modality, approach, or intervention you would rarely use

**1** = **Extremely Inappropriate**: A modality, approach, or intervention you would never use

Q17 Providers can choose from a variety of treatment modalities to involve clients and family members or support persons to varying degrees in treatment. Please rate the appropriateness of each of the following **MODALITIES** for clients age 18-25 with early psychosis and a comorbid trauma-related disorder. Please consider each modality separately.

|  | Extremely  Appropriate  9 | 8 | 7 | 6 | Equivocal  5 | 4 | 3 | 2 | Extremely  Inappropriate  1 |
| --- | --- | --- | --- | --- | --- | --- | --- | --- | --- |
| Individual Therapy: See the client alone (i.e., without family members or support persons) |  |  |  |  |  |  |  |  |  |
| Consultation: See family members or support persons alone (i.e., without client) |  |  |  |  |  |  |  |  |  |
| Conjoint or Family Therapy: See client and family members or support persons together |  |  |  |  |  |  |  |  |  |

Q18 Providers can choose from a variety of treatment approaches when treating clients who present with comorbid psychiatric conditions. Please rate the appropriateness of each of the following **APPROACHES** for clients age 18-25 with early psychosis and a comorbid trauma-related disorder. Please consider each approach separately.

|  | Extremely  Appropriate  9 | 8 | 7 | 6 | Equivocal  5 | 4 | 3 | 2 | Extremely  Inappropriate  1 |
| --- | --- | --- | --- | --- | --- | --- | --- | --- | --- |
| Single-Diagnosis: Treat psychotic symptoms only |  |  |  |  |  |  |  |  |  |
| Single-Diagnosis: Treat trauma symptoms only |  |  |  |  |  |  |  |  |  |
| Sequenced: Treat psychotic symptoms first; then treat trauma symptoms |  |  |  |  |  |  |  |  |  |
| Sequenced: Treat trauma symptoms first; then treat psychotic symptoms |  |  |  |  |  |  |  |  |  |
| Parallel: Treat psychotic symptoms and trauma symptoms at the same time, but with different providers |  |  |  |  |  |  |  |  |  |
| Integrated: Treat psychotic symptoms and trauma symptoms at the same time with the same providers |  |  |  |  |  |  |  |  |  |

**For each item, use scores in the 7-9 range to indicate a degree of appropriateness, scores in the 4-6 range to indicate a degree of equivocal opinion, or scores in the 1-3 range to indicate a degree of inappropriateness**, with the following anchor points:

**9** = **Extremely Appropriate**: Your modality, approach, or intervention of choice (you may have more than one per question)

**7-8** = **Appropriate**: A first-line modality, approach, or intervention you would often use

**4-6** = **Equivocal**: A second-line modality, approach, or intervention you would sometimes use (e.g., after first-line modalities, approaches, or interventions failed)

**2-3** = **Usually Inappropriate**: At most, a third-line modality, approach, or intervention you would rarely use

**1** = **Extremely Inappropriate**: A modality, approach, or intervention you would never use

The treatments providers use to address client symptoms are often comprised of a variety of interventions. The interventions listed below reflect those included in similar studies, but may not reflect the full range of interventions you (or members of your clinical staff) use to address psychotic or trauma symptoms in practice. Please help us to better understand the full range of interventions used by early psychosis programs by using the "Other" option to list and rate any additional interventions you would like to include in your response. Please [click here](https://qtrial2016q2.az1.qualtrics.com/CP/File.php?F=F_5hYPDWklGOvIjo9) to download definitions of the interventions listed for your reference.

Q19 Please rate the appropriateness of each of the following interventions for **ADDRESSING PSYCHOTIC SYMPTOMS** for clients with early psychosis and a comorbid trauma-related disorder. Please consider each intervention separately.

|  | Extremely  Appropriate  9 | 8 | 7 | 6 | Equivocal  5 | 4 | 3 | 2 | Extremely  Inappropriate  1 |
| --- | --- | --- | --- | --- | --- | --- | --- | --- | --- |
| Anxiety / Stress Management |  |  |  |  |  |  |  |  |  |
| Bilateral Stimulation |  |  |  |  |  |  |  |  |  |
| Case Management |  |  |  |  |  |  |  |  |  |
| Cognitive Restructuring |  |  |  |  |  |  |  |  |  |
| Emotion-Focused Strategies |  |  |  |  |  |  |  |  |  |
| Exposure Strategies |  |  |  |  |  |  |  |  |  |
| Interpersonal Effectiveness Training |  |  |  |  |  |  |  |  |  |
| Meditation / Mindfulness |  |  |  |  |  |  |  |  |  |
| Psychoeducation |  |  |  |  |  |  |  |  |  |
| Sensorimotor / Movement Strategies |  |  |  |  |  |  |  |  |  |
| Other (please specify) [Medium Text Box] |  |  |  |  |  |  |  |  |  |

Q20 Please rate the appropriateness of each of the following interventions for **ADDRESSING TRAUMA SYMPTOMS** for clients with early psychosis and a comorbid trauma-related disorder. Please consider each intervention separately.

|  | Extremely  Appropriate  9 | 8 | 7 | 6 | Equivocal  5 | 4 | 3 | 2 | Extremely  Inappropriate  1 |
| --- | --- | --- | --- | --- | --- | --- | --- | --- | --- |
| Anxiety / Stress Management |  |  |  |  |  |  |  |  |  |
| Bilateral Stimulation |  |  |  |  |  |  |  |  |  |
| Case Management |  |  |  |  |  |  |  |  |  |
| Cognitive Restructuring |  |  |  |  |  |  |  |  |  |
| Emotion-Focused Strategies |  |  |  |  |  |  |  |  |  |
| Exposure Strategies |  |  |  |  |  |  |  |  |  |
| Interpersonal Effectiveness Training |  |  |  |  |  |  |  |  |  |
| Meditation / Mindfulness |  |  |  |  |  |  |  |  |  |
| Psychoeducation |  |  |  |  |  |  |  |  |  |
| Sensorimotor / Movement Strategies |  |  |  |  |  |  |  |  |  |
| Other (please specify) [Medium Text Box] |  |  |  |  |  |  |  |  |  |

For Questions 21-24, you will be asked to **rate the appropriateness of trauma-focused treatment** **for clients age 18-25 with early psychosis and a comorbid trauma-related disorder** in general and under specific clinical and psychosocial conditions. Again, if your early psychosis program serves clients under age 18 or over age 25, you will have an opportunity to share how your responses would differ for these age groups later in the survey.

**Trauma-Focused Treatment** *includes treatments from a variety of theoretical orientations that address exposure to traumatic events directly by asking clients to recall or encounter thoughts, images, feelings, or situations related to traumatic events.*

**For each item, use scores in the 7-9 range to indicate a degree of appropriateness, scores in the 4-6 range to indicate a degree of equivocal opinion, or scores in the 1-3 range to indicate a degree of inappropriateness**, with the following anchor points:

**9** = **Extremely Appropriate**: Your treatment of choice (you may have more than one per question)

**7-8** = **Appropriate**: A first-line treatment you would often use

**4-6** = **Equivocal**: A second-line treatment you would sometimes use (e.g., after first-line treatments failed)

**2-3** = **Usually Inappropriate**: At most, a third-line treatment you would rarely use

**1** = **Extremely Inappropriate**: A treatment you would never use

Q21 Please rate the appropriateness of trauma-focused treatment for clients age 18-25 with early psychosis and a comorbid trauma-related disorder:

|  | Extremely  Appropriate  9 | 8 | 7 | 6 | Equivocal  5 | 4 | 3 | 2 | Extremely  Inappropriate  1 |
| --- | --- | --- | --- | --- | --- | --- | --- | --- | --- |
| Trauma-Focused Treatment |  |  |  |  |  |  |  |  |  |

Q22 Please rate the appropriateness of trauma-focused treatment for clients age 18-25 with early psychosis and a comorbid trauma-related disorder **AT EACH STAGE OF PSYCHOSIS**. Please consider each stage separately.

|  | Extremely  Appropriate  9 | 8 | 7 | 6 | Equivocal  5 | 4 | 3 | 2 | Extremely  Inappropriate  1 |
| --- | --- | --- | --- | --- | --- | --- | --- | --- | --- |
| Genetic Risk and Deterioration: Family history of psychosis and decline in functioning without attenuated or threshold psychotic symptoms |  |  |  |  |  |  |  |  |  |
| Ultra or Clinical High Risk: Attenuated psychotic symptoms |  |  |  |  |  |  |  |  |  |
| First-episode psychosis: Onset of threshold psychotic symptoms less than 5 years ago |  |  |  |  |  |  |  |  |  |
| Established or Chronic Psychosis: Onset of threshold psychotic symptoms more than 5 years ago |  |  |  |  |  |  |  |  |  |

**For each item, use scores in the 7-9 range to indicate a degree of appropriateness, scores in the 4-6 range to indicate a degree of equivocal opinion, or scores in the 1-3 range to indicate a degree of inappropriateness**, with the following anchor points:

**9** = **Extremely Appropriate**: Your treatment of choice (you may have more than one per question)

**7-8** = **Appropriate**: A first-line treatment you would often use

**4-6** = **Equivocal**: A second-line treatment you would sometimes use (e.g., after first-line treatments failed)

**2-3** = **Usually Inappropriate**: At most, a third-line treatment you would rarely use

**1** = **Extremely Inappropriate**: A treatment you would never use

Assume you (or a member of your clinical staff) are considering the use of trauma-focused treatment for a client age 18-25 with early psychosis and a comorbid trauma-related disorder that you consider stable, but you are concerned about increasing symptom severity or contributing to client crisis or hospitalization.

Q23 Please rate the appropriateness of proceeding with trauma-focused treatment for clients age 18-25 with early psychosis and a comorbid trauma-related disorder **UNDER THE FOLLOWING CURRENT CONDITIONS**. Please consider each condition separately.

|  | Extremely  Appropriate  9 | 8 | 7 | 6 | Equivocal  5 | 4 | 3 | 2 | Extremely  Inappropriate  1 |
| --- | --- | --- | --- | --- | --- | --- | --- | --- | --- |
| Significant Life Stressors |  |  |  |  |  |  |  |  |  |
| Low Involvement of Family Members or Support Persons |  |  |  |  |  |  |  |  |  |
| Attenuated or Residual Psychotic Symptoms |  |  |  |  |  |  |  |  |  |
| Comorbid Personality Disorder |  |  |  |  |  |  |  |  |  |
| Other Comorbid Psychiatric Disorder |  |  |  |  |  |  |  |  |  |
| Other (please specify) [Medium Text Box] |  |  |  |  |  |  |  |  |  |

Q24 Please rate the appropriateness of proceeding with trauma-focused treatment for clients age 18-25 with early psychosis and a comorbid trauma-related disorder **UNDER THE FOLLOWING PAST CONDITIONS**. Please consider each condition separately.

|  | Extremely  Appropriate  9 | 8 | 7 | 6 | Equivocal  5 | 4 | 3 | 2 | Extremely  Inappropriate  1 |
| --- | --- | --- | --- | --- | --- | --- | --- | --- | --- |
| Exposure to Single Traumatic Event |  |  |  |  |  |  |  |  |  |
| Exposure to Multiple Traumatic Events |  |  |  |  |  |  |  |  |  |
| Severe Symptoms When Symptomatic |  |  |  |  |  |  |  |  |  |
| Long-Duration Symptoms When Symptomatic |  |  |  |  |  |  |  |  |  |
| Poor Functioning When Symptomatic |  |  |  |  |  |  |  |  |  |
| Hospitalization |  |  |  |  |  |  |  |  |  |
| High Violence Risk |  |  |  |  |  |  |  |  |  |
| High Suicide Risk |  |  |  |  |  |  |  |  |  |
| Non-Suicidal Self-Injurious Behavior (NSSI) |  |  |  |  |  |  |  |  |  |
| Substance Use |  |  |  |  |  |  |  |  |  |
| Other (please specify) [Medium Text Box] |  |  |  |  |  |  |  |  |  |

Q25 Does your early psychosis program serve clients under age 18?

- Yes
- No

If No Is Selected, Then Skip To Q27

Q26 Would any of your responses about appropriate approaches, modalities, interventions, or treatments for clients with early psychosis and a comorbid trauma-related disorder differ for **CLIENTS UNDER AGE 18**?

- Yes (please describe) [Large Text Box]
- No

Q27 Does your early psychosis program serve clients over age 25?

- Yes
- No

If No Is Selected, Then Skip To Q29

Q28 Would any of your responses about appropriate approaches, modalities, interventions, or treatments for clients with early psychosis and a comorbid trauma-related disorder differ for **CLIENTS OVER AGE 25**?

- Yes (please describe) [Large Text Box]
- No

Q29 Are you aware of any **BARRIERS** your early psychosis program has encountered in attempting to treat clients with early psychosis and a comorbid trauma-related disorder?

- Yes (please describe) [Large Text Box]
- No

Q30 Please provide any other information you think would help to improve the treatment of clients with early psychosis and a comorbid trauma-related disorder in early psychosis programs: [Essay Text Box]

**Definitions of Key Terms in Survey**

**Early Psychosis** — Onset of threshold psychotic symptoms less than 5 years ago

**Psychotic Symptoms** — Delusions (e.g., paranoid, grandiose, or somatic ideas or beliefs that are firmly held despite contrary evidence), hallucinations (e.g., auditory, visual, somatic, olfactory, or gustatory perceptions in the absence of corresponding external stimuli), or disorganized communication (e.g., losing track of or jumping around from one topic to another in conversation; behaving in ways that do not fit the situation)

**Trauma-Related Disorder** — Posttraumatic stress disorder (PTSD) or other psychiatric disorder resulting from exposure to one or more traumatic event

**Traumatic Event** — A situation (e.g., child physical, sexual, emotional, or psychological abuse; child neglect; domestic, school, or community violence; natural disasters like fire, tornado, floor, or hurricane; vehicular or other serious accident; war, terrorism, or refugeeism; medical trauma; and traumatic grief like sudden and/or violence death of a loved one) in which an individual is exposed directly or indirectly (e.g., witnessing, learning about) to actual or threatened death, serious injury, or sexual violence

**Trauma Symptoms** — Re-experiencing or intrusion symptoms (e.g., recurrent, involuntary, or distressing thoughts or images of the traumatic event), avoidance symptoms (e.g., avoidance of thoughts of, feelings about, and reminders associated with the traumatic event), negative cognitions or mood (e.g., persistent, distorted, or exaggerated negative beliefs about self, others, world, or cause or consequences of the traumatic event accompanied by negative emotions or inability to experience positive emotions), and hyperarousal symptoms (e.g., impaired emotional, behavioral, or cognitive regulation)

**Definitions of Interventions in Survey**

**Anxiety/Stress Management** – A broad class of techniques that focus on the development of coping skills to reduce stress and stress-related difficulties such as muscle ache, rumination, or poor sleep. Techniques include muscle relaxation training, focused breathing or breathing retraining, or sleep hygiene.

**Bilateral Stimulation** – A class of techniques which include the presence of alternating attention and stimulation such as eye movements which track the back and forth of a visual stimulus (e.g., therapist finger) or through other stimuli such as a tone or tap on body while the individual thinks about or imagines troubling memories. The purpose of this intervention is to desensitize the individual to troublesome thoughts, images, or memories and to reduce overall distress.

**Case Management** – The coordination of services and resources to benefit the client. This includes medication, employment training, housing, day treatment, or HIV testing.

**Cognitive Restructuring** – Interventions designed to help individuals alter their understanding of the meaning of their experiences. Techniques include exploring and revising identified maladaptive cognitions or reappraising the meaning of an event or experience.

**Emotion-Focused Strategies** – Techniques that focus attention to and awareness of the individual’s emotional experiences for the purposes of clarifying meaning and enhancing appraisal of past, ongoing, and future events and to help guide actions and decisions. Emotion regulation interventions focus on improving the individual’s ability to manage, modify, and express emotions within a range that optimizes achievement of goals.

**Exposure Strategies** – Individuals remember and describe the thoughts, images, or feelings or encounter situations associated with troublesome events or experiences for the purposes of tolerating and reducing the distress associated with the memory. This usually, but not always, includes a reappraisal and revision of the meaning of the events or experiences.

**Interpersonal Effectiveness Training** – Interventions focus on improving social skills, identifying and resolving interpersonal difficulties in relationships of various kinds (e.g., work, social, and intimate relationships) and strengthening positive interpersonal and relational expectations.

**Meditation/Mindfulness** – Interventions in which directed attention is given to a single stimulus such as one’s breath, a sound, or a light for a sustained period of time for the purposes of reducing physical and mental stress and improving concentration and sense of well-being. Mindfulness is a class of techniques which draws attention to a variety of subjective experiences such as feelings and sensations without judgment or action with the goal of reducing distress and anxiety and enhancing sense of well being.

**Psychoeducation** – Systematic description to clients and their significant others about symptoms and education about treatments (e.g., rationale, efficacy). The goal of psychoeducation is to provide support to clients by expressing understanding and familiarity with client’s problems and by reassuring clients that symptoms and problems can be overcome with time and treatment.

**Sensorimotor/Movement Strategies** – Interventions that focus on bodily sensations and movement to address and resolve troublesome memories in a nonverbal fashion and to improve attention, decrease dissociation, and increase energy and sense of the experience of bodily integration.

# Supplementary Table 1. *Early psychosis programs in the United States surveyed as part of this study*

| **State** | **Early Psychosis Program** |
| --- | --- |
| Arizona | Early Psychosis Intervention Center (EPICENTER) |
| California | Aftercare Research Program |
|  | PREP/BEAM San Francisco |
|  | PREP/BEAM San Mateo |
|  | PREP Monterey |
|  | PREP Alameda |
|  | Cognitive Assessment and Risk Evaluation (CARE) Program |
|  | Early Diagnosis and Preventive Treatment (EDAPT) Clinic |
|  | First Hope |
|  | INSPIRE |
|  | Kickstart |
|  | LIFE Path |
|  | Momentum for Mental Health |
|  | Orange County Center for Resiliency Education and Wellness (OC CREW) |
|  | Prevention / Early Intervention Services for Transition-Age Youth |
|  | Prodrome Assessment Research and Treatment (PART) Program |
|  | Stanford Early Psychosis Clinic (INSPIRE Clinic) |
|  | Starlight Community Services |
|  | Supportive Outreach & Access to Resources (SOAR) -- Napa County |
|  | Supportive Outreach & Access to Resources (SOAR) -- Solano County |
|  | Telecare Early Intervention and Recovery (TEIR) Program |
|  | The Staglin Music Festival Center for the Assessment and Prevention of Prodomal States (CAPPS) |
|  | UCSF Early Psychosis Clinic |
|  | Ventura Early Prevention Services (VIPS) |
| Colorado | Adolescent Treatment and Preventive Development (ADAPT) |
| Connecticut | Prevention through Risk Identification, Management & Education (PRIME) Research Clinic |
|  | Specialized Treatment in Early Psychosis (STEP) |
|  | The Early Psychosis Program at the POTENTIAL Outpatient Clinic |
| Florida | EPIC Program |
|  | NAVIGATE Team |
| Georgia | Emory Mental Health & Development Program |
|  | Prevention and Early Intervention Program |
| Illinois | ADAPT Chicago |
|  | First Episode Psychosis Program at University of Illinois Medical Center |
| Indiana | Prevention and Recovery Center for Early Psychosis (PARC) |
| Iowa | First Episode Recovery Support Team (FERST) |
|  | RESTORE Program (Eyerly Ball) |
| Maine | Portland Identification and Early Referral (PIER) Mental Health Attitudes of Youth (MAY) Study |
| Maryland | Strive for Wellness Clinic |
|  | MPRC First Episode Clinic (FEC) |
|  | RAISE Connection Program |
|  | Johns Hopkins Early Psychosis Intervention Clinic (EPIC) / Maryland EIP (EPIC/EIP) |
|  | OnTrack Maryland at Family Services, Inc. |
| Massachusetts | Center for Early Detection, Assessment, & Response to Risk (CEDAR) |
|  | First-Episode and Early Psychosis Program (FEPP) |
|  | Prevention and Recovery in Early Psychosis (PREP) |
|  | Prevention and Recovery in Early Psychosis (PREP) West |
|  | Screening and Treatment of Early Psychosis (STEP) Clinic |
|  | The Collaborative Pathway |
| Michigan | Early Treatment and Cognitive Health (ETCH) |
|  | NAVIGATE Team of Easter Seals Michigan |
|  | NAVIGATE Team of InterAct of Michigan |
| Minnesota | First-Episode Early Psychosis Program (FEPP) |
| Mississippi | NAVIGATE-Enhanced PACT Team |
| Missouri | First Contact Assessment Service |
| Montana | NAVIGATE-Enhanced TAY Team |
| New Mexico | Early Assessment and Resource Linkage for Youth (EARLY) Consultation Clinic |
| New York | Early Treatment Program (ETP)/OnTrackNY Lenox Hill Hospital |
|  | Early Treatment Program (ETP) Zucker Hillside Hospital |
|  | Kings OnTrackNY |
|  | NAVIGATE Team of the Adult, Child, and Family Clinic |
|  | OnTrack at Lake Shore Behavioral Health, Inc. |
|  | OnTrack NY Central New York (CNY) |
|  | OnTrack NY@MHA (Westchester) |
|  | OnTrack NY@Parsons |
|  | OnTrackNY at Jewish Board |
|  | OnTrackNY Rochester |
|  | OnTrackNY Suffolk |
|  | OnTrackNY@Bellevue |
|  | Recognition and Prevention (RAP) Program |
|  | The Center of Prevention & Evaluation (COPE) |
|  | The Lieber Schizophrenia Research Clinic (LSRC) |
|  | Washington Heights Community Service |
| North Carolina | Outreach and Intervention Support Services (OASIS) -- Carrboro |
|  | Outreach and Intervention Support Services (OASIS) -- Raleigh |
|  | Prevention through Risk Identification, Management & Education (PRIME) Study |
| Ohio | Early Psychosis Intervention Center (EPICENTER) Ohio |
|  | FIRST Cuyahoga County |
|  | FIRST Greater Cincinnati (Greater Cincinnati Behavioral Health Services) |
|  | FIRST Greater Lima |
|  | FIRST Lucas & Wood Counties |
|  | FIRST Mahoning County |
|  | FIRST Portage County |
|  | FIRST Stark County |
|  | FIRST Trumbull County |
|  | FIRST Summit County |
|  | The Best Practices in Schizophrenia Treatment (BeST) Center |
| Oklahoma | NAVIGATE Program of HOPE Community Services |
| Oregon | EASA Baker County |
|  | EASA Center for Excellence |
|  | EASA Center for Human Development, Inc. (Union County) |
|  | EASA Clatsop Behavioral Healthcare |
|  | EASA Columbia Community Mental Health |
|  | EASA Community Health Alliance (Douglas County) |
|  | EASA Deschutes Co. Child & Family Program |
|  | EASA Jackson County Mental Health |
|  | EASA Klamath Basin Behavioral Health |
|  | EASA Life Works NW Beaverton (Washington County) |
|  | EASA Life Works NW Milwaukie (Clackamas County) |
|  | EASA Lifeways, Inc. (Malheur County) |
|  | EASA Lifeways, Inc. Hermiston (Umatilla County) |
|  | EASA Lifeways, Inc. Pendleton (Umatilla County) |
|  | EASA Linn Co. Mental Health |
|  | EASA Marion Co. Children's Behavioral Health |
|  | EASA Mid-Columbia Center for Living -- Hood River |
|  | EASA Mid-Columbia Center for Living -- The Dalles |
|  | EASA Multnomah Co. Mental Health & Addication Services |
|  | EASA Options Creekside Center |
|  | EASA Peace Health Oregon / Lane County Behavioral Health Services |
|  | EASA Polk County Mental Health |
|  | EASA Rockwood Community Health Center (Multnomah County) |
|  | EASA Tillamooka Family Counseling Center |
|  | EASA Wallowa Valley Center for Wellness |
|  | EASA Yamhill Co. Adult Mental Health |
| Pennsylvania | Penn Psychosis Evaluation and Recovery Center (PENN PERC) |
|  | Psychosis Education, Assessment, Care, and Empowerment (PEACE) |
| Texas | First Episode Psychosis Program (FEPP) |
| Utah | Weber Human Services |
| Virginia | NAVIGATE Team of Highlands Community Services |
|  | Turning Point |
| Washington | New Journeys Early Intervention Project |

Supplementary Table 2. *States represented by survey participants*

| **State** | **%** | **N** |
| --- | --- | --- |
| California | 24 | 12 |
| New York | 12 | 6 |
| Ohio | 12 | 6 |
| Oregon | 12 | 6 |
| Massachusetts | 6 | 3 |
| Connecticut | 4 | 2 |
| Georgia | 4 | 2 |
| Pennsylvania | 4 | 2 |
| Florida | 2 | 1 |
| Illinois | 2 | 1 |
| Maine | 2 | 1 |
| Maryland | 2 | 1 |
| Michigan | 2 | 1 |
| Mississippi | 2 | 1 |
| Missouri | 2 | 1 |
| Oklahoma | 2 | 1 |
| Utah | 2 | 1 |
| Virginia | 2 | 1 |

Supplementary Table 3. *Types of services offered as part of early psychosis care, as reported by participants in the survey*

| **Service Offered** | **%** | **N** |
| --- | --- | --- |
| Family Psychoeducation and Support | 100 | 49 |
| Comprehensive Assessment | 98 | 48 |
| Individual Psychotherapy | 94 | 46 |
| Medication Management | 94 | 46 |
| Care Coordination | 92 | 45 |
| Supported Education and/or Employment Services | 84 | 41 |
| Case Management | 82 | 40 |
| Group Psychotherapy | 59 | 29 |
| Other | 46 | 23 |

Supplementary Table 4. *Participant and program training and experience in early psychosis and trauma treatment*

|  | **Participants** | | **Programs** | |
| --- | --- | --- | --- | --- |
| **Early Psychosis Treatment** | **Formal  Training (N)** | **Supervised Clinical Experience (N)** | **Formal  Training (N)** | **Supervised Clinical Experience (N)** |
| Cognitive Behavioral Therapy for Psychosis | 23 | 9 | 22 | 16 |
| Multi-Family Group | 14 | 6 | 13 | 10 |
| Early Assessment and Support Alliance | 6 | 2 | 5 | 3 |
| NAVIGATE | 5 | 2 | 5 | 1 |
| Cognitive Behavioral Therapy | 4 | 4 | 6 | 6 |
| Family Focused Therapy | 3 | 3 | 2 | 2 |
| FIRST Model | 3 | 2 | 2 | 1 |
| Motivational Interviewing | 3 | 0 | 3 | 2 |
| Family Psychoeducation | 2 | 0 | 1 | 1 |
| Individual Resiliency Training | 2 | 1 | 4 | 1 |
| OnTrackNY | 2 | 2 | 2 | 2 |
| Open Dialogue | 2 | 1 | 2 | 1 |
| PIER Model | 2 | 1 | 2 | 1 |
| Social Cognition and Interaction Training | 2 | 2 | 2 | 2 |
| Acceptance & Commitment Therapy | 1 | 0 | 0 | 0 |
| Cognitive Therapy | 1 | 0 | 0 | 0 |
| Individualized Placement and Support | 1 | 0 | 2 | 1 |
| Integrative Dual Diagnosis Treatment | 1 | 1 | 0 | 1 |
| Intentional Peer Support | 1 | 0 | 1 | 0 |
| Narrative Therapy | 1 | 1 | 0 | 0 |
| Recovery-Oriented Cognitive Therapy for Psychosis | 1 | 0 | 1 | 0 |
| Solution Focused Therapy | 1 | 0 | 1 | 1 |
| Supportive Interpersonal Therapy | 1 | 1 | 1 | 1 |
| Voice Dialogue | 1 | 0 | 0 | 0 |
| Need-Adapted Treatment | 0 | 1 | 1 | 0 |
| Cognitive Remediation | 0 | 0 | 2 | 1 |
| Insight Oriented Therapy | 0 | 0 | 1 | 1 |

|  | **Participants** | | **Programs** | |
| --- | --- | --- | --- | --- |
| **Trauma Treatment** | **Formal  Training (N)** | **Supervised Clinical Experience (N)** | **Formal  Training (N)** | **Supervised Clinical Experience (N)** |
| Trauma-Focused Cognitive Behavioral Therapy | 12 | 3 | 7 | 5 |
| Dialectical Behavior Therapy | 6 | 1 | 2 | 1 |
| Cognitive Behavioral Therapy | 4 | 2 | 2 | 2 |
| Cognitive Processing Therapy | 3 | 1 | 1 | 2 |
| Mindfulness Based Stress Reduction | 3 | 0 | 0 | 0 |
| Narrative Therapy | 3 | 2 | 1 | 1 |
| Sanctuary Model | 3 | 0 | 0 | 0 |
| Eye Movement Desensitization and Reprocessing | 2 | 1 | 0 | 0 |
| Collaborative Problem Solving | 2 | 0 | 0 | 0 |
| Prolonged Exposure Therapy | 2 | 0 | 0 | 1 |
| Seeking Safety | 2 | 2 | 1 | 0 |
| Acceptance & Commitment Therapy | 1 | 0 | 0 | 0 |
| Attachment, Self-Regulation, and Competency (ARC) | 1 | 1 | 0 | 0 |
| Brief Therapy for Trauma-Related Disorders | 1 | 0 | 0 | 0 |
| Child-Parent Psychotherapy (CPP) | 1 | 0 | 0 | 0 |
| Cognitive Reprocessing | 1 | 1 | 0 | 0 |
| Cognitive Therapy | 1 | 2 | 0 | 0 |
| Motivational Interviewing | 1 | 0 | 1 | 0 |
| Sensorimotor Psychotherapy | 1 | 0 | 0 | 0 |
| Somatic Experiencing | 1 | 0 | 0 | 0 |
| Trauma and Grief Component Therapy for Adolescents | 1 | 0 | 0 | 0 |
| Trauma Recovery Empowerment Model | 1 | 1 | 0 | 0 |
| Psychodynamic Psychotherapy | 0 | 1 | 0 | 0 |
| Collaborative Problem Solving | 0 | 0 | 1 | 1 |
